# Mild Cognitive Impairment Progression and Alzheimer’s Disease Risk: A Comprehensive Analysis of 3553 Cases over 203 Months

**DOI:** 10.3390/jcm13020518

**Published:** 2024-01-17

**Authors:** Nevra Öksüz, Reza Ghouri, Bahar Taşdelen, Derya Uludüz, Aynur Özge

**Affiliations:** 1 Department of Neurology, School of Medicine, Mersin University, Mersin 33110, Turkey; nvrksz@gmail.com (N.Ö.); dr.rezaghouri@gmail.com (R.G.); 2 Department of Biostatistics, School of Medicine, Mersin University, Mersin 33110, Turkey; bahartasdelen@gmail.com; 3 Department of Neurology, Brain 360 Holistic Approach Center, İstanbul 34353, Turkey; deryaulu@yahoo.com

**Keywords:** mild cognitive impairment, cognitive function, disease progression, dementia, hippocampal atrophy, Alzheimer’s disease, Non-AD MCI

## Abstract

This study aimed to elucidate the long-term progression of mild cognitive impairment (MCI) within a comprehensive longitudinal dataset, distinguish it from healthy aging, explore the influence of a dementia subtype on this progression, and identify potential contributing factors. Patients with prodromal and preclinical cases underwent regular neuropsychological assessments utilizing various tools. The study included a total of 140 participants with MCI, categorized into Alzheimer’s disease (AD) and non-AD subtypes. Our dataset revealed an overall progression rate of 92.8% from MCI to the clinical stage of dementia during the follow-up period, with an annual rate of 15.7%. Notably, all prodromal cases of Lewy body dementia/Parkinson’s disease (LBD/PDD) and frontotemporal dementia (FTD) advanced to clinical stages, whereas 7% of vascular dementia (VaD) cases and 8.4% of AD cases remained in the prodromal stage throughout follow-up. Furthermore, we observed a faster progression rate in MCI-AD cases compared to non-AD sufferers (53.9% vs. 35.5%, Entropy: 0.850). This study revealed significant cognitive changes in individuals with MCI over time. The mini-mental state examination (MMSE), global deterioration scale (GDS), and calculation tests were the most effective tests for evaluation of MCI. These findings may offer valuable insights for the development of personalized interventions and management strategies for individuals with MCI.

## 1. Introduction

Dementia represents a progressive decline in cognitive function attributed to brain damage or disease and is among the leading causes of elderly disability worldwide. Currently, there is significant interest in prodromal dementia, the stage of cognitive decline preceding the formal diagnosis of dementia. This stage is characterized by subtle shifts in memory, thinking, mood, and behavior, detectable through comprehensive assessments and neuropsychological tests [1].

Mild cognitive impairment (MCI), also known as “preclinical dementia”, describes a condition marked by cognitive decline exceeding what is expected for one’s age and education level but not meeting the criteria for dementia. Individuals with MCI may experience challenges with memory, language, judgment, and other cognitive functions [2]. Ongoing research is actively exploring early detection and management strategies for prodromal dementia, with significant implications for enhancing dementia diagnosis and treatment among the elderly. These studies also aim to identify early or late progression cases and potential contributing factors [3].

Factors potentially influencing the risk of MCI advancing to clinical dementia include age (with increased age posing greater risk), MCI subtype (amnestic MCI carries a higher risk of progressing to dementia than non-amnestic MCI), Apolipoprotein E (APOE) genotype (the APOE e4 allele is associated with an elevated risk of MCI progressing to Alzheimer’s disease (AD)), performance on neuropsychological tests, brain imaging characteristics like reduced volume or increased white matter intensities (WMI), cerebrospinal fluid (CSF) biomarkers, and a lack of social engagement and cognitive stimulation [4]. Dementia progression, encompassing prodromal and preclinical stages, varies across different types of dementia. For instance, Alzheimer’s disease typically evolves slowly over several years, with symptoms deteriorating gradually. In contrast, vascular dementia (VaD) may manifest with a swifter onset and progression, often stemming from a stroke or vascular event. Frontotemporal dementia (FTD) tends to advance more rapidly than AD, with symptoms worsening over months to years. It is important to note that not all individuals with MCI will progress to dementia, and there is no definitive way to predict which individuals will. Regular monitoring and risk factor management can help slow MCI’s progression to dementia and enhance patients’ quality of life. However, the current understanding does not provide a consensus on which cases are more likely to advance to clinical phases during the preclinical stages, leaving clinicians without specific prognostic information to share with patients [2,4].

This study mainly focused on the following endpoints:

- Define the long-term progression of MCI in a comprehensive longitudinal follow-up data set under the same senior author’s supervision.
- Differentiate from the healthy elders.
- Define the effect of the type of dementia on the process.
- Define the potential cofactors on the progression to project some clues for further studies.

## 2. Materials and Methods

### 2.1. Data Collection and Patient Selection

Patients in this study were recruited from a dementia database comprising individuals who had sought consultation at the Dementia Outpatient Clinic of the Neurology Department at the Mersin University Medical Faculty in Turkey between 2000 and 2022, under the supervision of the same senior author (AO). The dataset included individuals from the following groups.

Healthy Controls: Healthy controls consisted of spouses and volunteer relatives of hospital staff, often living in the same social and biological environment as the patients but not related to them, as stated in the methods section. The inclusion criteria were as follows:

- Over 18 years of age and under 85 years of age.
- To have signed the voluntary consent form.
- No known neurodegenerative, toxic, metabolic, malignant, etc. diseases requiring treatment.
- No recent history of head trauma or history of recurrent head trauma in the past.
- No major psychiatric disorder requiring treatment for more than 6 months in the past.
- No history of alcohol or substance abuse.

As with the patients, routine biochemistry and brain imaging were requested from the controls. These individuals also had at least 3 follow-up visits with neuropsychological tests, and those who were diagnosed with MCI or dementia were reported.

Prodromal Dementia: This group primarily consists of the relatives of dementia patients who come for routine check-ups and have received a prodromal dementia diagnosis following assessments. Within this group, there are individuals with obvious episodic memory loss, a decrease in problem-solving and decision-making abilities, and those who repeatedly ask questions.

Preclinical Dementia or MCI cases: In this group, there are Mild Cognitive Impairment (MCI) patients with subtle episodic memory loss, a decrease in learning abilities, impaired executive functions, and delayed recall.

Clinical Dementia Cases: Patients with severe episodic memory loss, significantly impaired learning abilities, pronounced deficits in recall, difficulty finding words, orientation disturbances, functionality levels, and marked impairments in executive functions, such as problem-solving, decision-making, and judgment, have been included.

This study primarily focused on prodromal and preclinical cases, with MCI and prodromal dementia diagnosed according to the revised Petersen criteria and MCI Continuum suggestions [4,5]. Ethical approval and institutional permission for the study were obtained by the Toros University Ethics Committee (decision no: 2023/28, date: 10 March 2023).

All patients underwent regular quarterly visits at the same clinic under the supervision of the same author (AO). Neuropsychological evaluations were generally conducted every other visit (approximately 2 times per year) and recorded in the database. Additionally, patients had several other visits related to medical or medication-related symptoms within the scope of this study. During each visit, physicians provided medication and necessary laboratory tests, as needed. Patients underwent differential diagnosis, neurological examinations, and neuropsychological examinations using a consistent methodology. Data were recorded in the electronic database of the Turkish Alzheimer’s Working Group (www.epikriz.com/dementiadataset) to ensure comprehensive neuropsychological evaluation, covering various cognitive domains, functional capacity, cognition, numerical range, calculation, abstraction, Word Memory Test (WMT), Clock Drawing Test (CDT), and GDS. Methodologies for these tests have been detailed previously [6,7], ensuring assessment consistency and accuracy.

To distinguish MCI from other causes of dementia, a neuroimaging protocol with magnetic resonance imaging (MRI) or computerized tomography (CT) was employed for differential diagnosis. The assessment of white matter hyperintensities, as quantified by the Fazekas score, was integral to our evaluation of cerebral small vessel disease. This scoring system, initially proposed by Fazekas et al., classifies the severity of white matter changes on neuroimaging, providing valuable insights into the vascular pathology associated with cognitive impairment [8]. Our examination of hippocampal atrophy involved a systematic grading system, as outlined by Scheltens scale. This scale allows for a standardized and reliable characterization of hippocampal volume loss, aiding in the differential diagnosis of neurodegenerative diseases. The choice of this grading system was informed by its widespread acceptance and validation in the literature [9,10,11]. Some cases underwent standardized single photon emission computed tomography (SPECT) or positron emission tomography (PET) investigations when necessary. The inclusion of the Barthel Daily Living Activities Scale (BDLAS) was pivotal in our study, offering a comprehensive evaluation of the functional independence and activities of daily living in our patient cohort. Originally introduced by Mahoney and Barthel, this widely accepted scale has demonstrated reliability and validity in assessing functional abilities across various health conditions and was chosen for its suitability in capturing nuanced changes in daily functioning associated with cognitive decline [12,13].

Exclusion criteria encompassed known inflammatory, infectious, or immune diseases causing cognitive disturbances, overlapping syndromes (e.g., AD plus VaD, motor neuron disorders), comorbid neuropsychiatric disorders (e.g., previously diagnosed psychotic disorders, dependency), significant head trauma, severe renal or hepatic failure, recent severe hemodynamic disturbances (e.g., decompensated heart failure, shock, acute myocardial ischemia), residence in nursing homes or palliative care units, and patient or legal representative refusal to participate in the study.

### 2.2. Statistical Analysis

Demographic and some clinical characteristics (such as symptoms, comorbidities, and family history of dementia and vascular disease) were compared using one-way ANOVA (for age and follow-up) and chi-squared tests. Neuropsychiatric assessment of cases with MCI was performed using repeated measures of ANOVA for normally distributed variables. Otherwise, Friedman ANOVA was used to assess neuropsychiatric change. Proportional differences between males and females in ApoE, hippocampal atrophy, Fazekas grading, hydrocephalus, epilepsy, and extrapyramidal symptoms were compared using chi-squared or likelihood ratio test.

In this study, our main aim was to define the long-term course of MCI and to disentangle the effect of the type of dementia on admission. We used group-based trajectory models to identify clusters of slowly and rapidly progressing patients according to their outcome measures. Entropy-based goodness-of-fit statistics were used to assess the ability of the model to classify subgroups, with entropy values greater than 0.70 indicating acceptable classification [14]. The significance of the outcome measures was assessed using their model coefficients (±standard errors) and *p*-values. A value of *p* < 0.05 was considered statistically significant.

We also summarized the progression from admission to last visit descriptively. The analysis of group-based progression modelling was performed using the STATA plugin, while the other analyses were performed using STATISTICA 13.0. We also summarized the progression from admission to last visit descriptively.

## 3. Results

**Figure 1:**
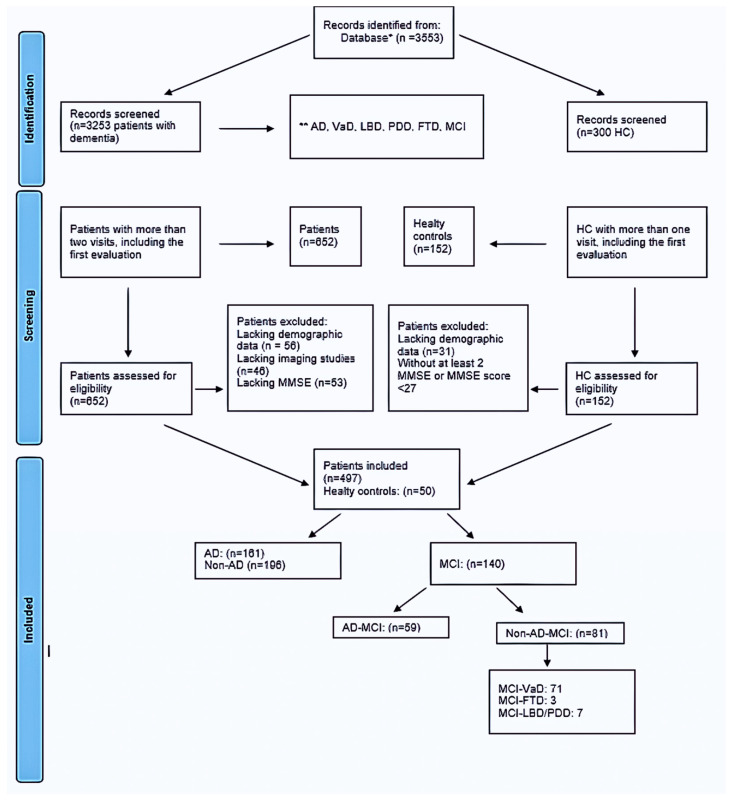
Flow chart of study sample. * Epikriz.com (Turkish Alzheimer Database, Mersin Branch). ** AD: Alzheimer’s disease, VaD: Vascular dementia, LBD: Lewy body dementia, PDD: Parkinson’s disease with dementia, FTD: Frontotemporal dementia, MCI: Mild cognitive impairment, MMSE: mini-mental state examination, HC: Healthy controls.

Evaluation of MCI Cases: In this study, we assessed a total of 140 cases (Figure 1) selected from a dementia database. The majority of cases belonged to the MCI-VaD category (n = 71, 50.7%), followed by AD (59 cases, 42.1%), while FTD and Lewy body dementia/Parkinson’s disease (LBD/PDD) categories had lower frequencies, with counts of three and seven, respectively.

The study examined the characteristics of participants with various MCI subtypes, including healthy controls (Table 1). Among the 140 participants, representing 73.68% of the total sample size of 190, the mean age varied across groups. Healthy controls had a mean age of 65.59 ± 9.96 years, while the MCI-AD group averaged 69.51 ± 6.32 years. The Non-AD MCI group was the oldest, with a mean age of 71.12 ± 7.32 years (*p* = 0.0015). The mean age for the entire MCI group was 70.44 ± 6.94 years. The follow-up duration at the first, second, and last visits differed among groups, with the longest follow-up duration for the healthy control group at the first visit (mean: 51.69 ± 107.05 months) and the shortest for the MCI-AD group at the second visit (mean: 26.72 ± 27.58 months). However, there were no significant differences in follow-up duration between the groups, with a maximum follow-up duration of 203 months.

Regarding gender, most participants were male, except for the healthy control group, where 56% were female. Educational levels varied, with the majority having basic school education (ranging from 79.66% to 90.12%) across different groups. Most participants lived with family members, and the most commonly reported symptoms were language dysfunction and memory dysfunction, particularly prominent in the MCI-AD group (64.41% and 44.07%, respectively). Behavioral problems, self-care issues, and incontinence were the least reported symptoms in patients diagnosed with clinical dementia in the process.

Hypertension and hyperlipidemia were the most common comorbidities, with prevalence rates ranging from 18% to 46.92% and 18% to 24.69%, respectively, across different patient groups. Conversely, thyroid dysfunction, stroke, and current smoking were less prevalent. The percentage of regular alcohol consumption was low in all patient groups, while a significant percentage of patients reported quitting smoking and alcohol consumption. Notably, a significant percentage of participants had a history of epilepsy and extrapyramidal symptoms in the process, with the highest prevalence observed in the Non-AD MCI group. The table also reports family history, with the percentage of individuals having a family history of vascular disease increasing over time.

Cognitive tests administered over three visits (Table 2) showed significant changes in scores over time. The Barthel Daily Living Activities Scale (BDLAS) scores increased significantly from the first to the third visit (*p* = 0.00042), while Elderly Daily Living Activity Scale (EDLAS) scores decreased significantly (*p* = 0.00663). Mini-mental state examination (MMSE) scores exhibited a significant decline (*p* < 0.0001). Calculation, Global Deterioration Scale (GDS), and MMSE scores were the most favorable for overall MCI evaluation (*p* < 0.0001). Other scores remained consistent across the three visits, with significant improvements in Word memory test-2 (WMT-2) and WMT-recall scores (*p*-values of 0.00146 and 0.00362, respectively).

In terms of laboratory biomarkers, the APOE genotype distribution showed no significant gender-based differences. Most individuals had the E3/E4 genotype, followed by E3/E3. For hippocampal atrophy, most males and females had Grade 1 atrophy, with no significant gender-based differences. Fazekas grades displayed no significant differences between males and females. Epilepsy was more prevalent among females, while extrapyramidal symptoms were more common among males, with no significant gender-based differences (Table 3)

**Figure 2:**
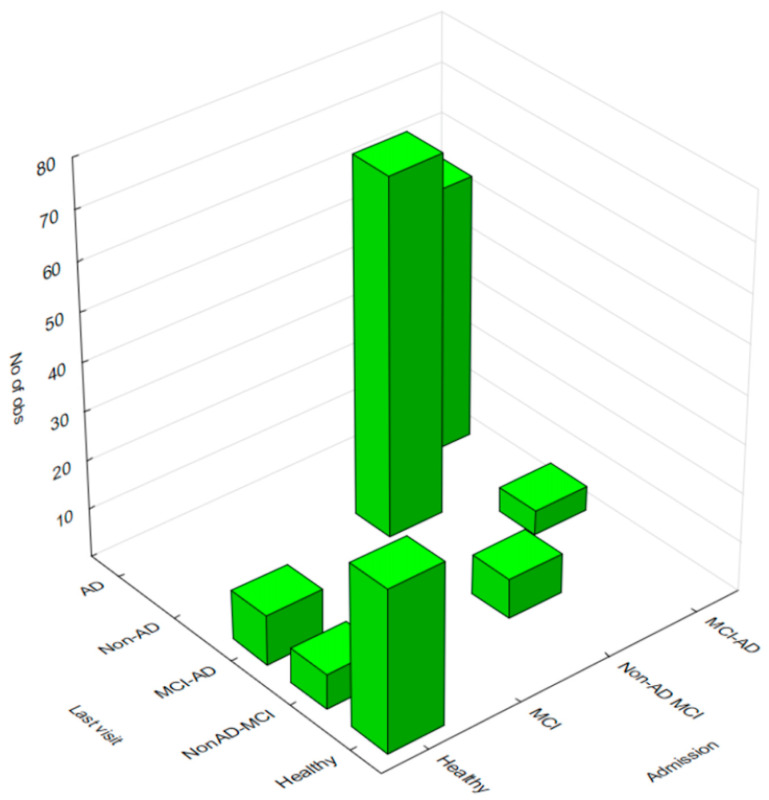
Progression of the cases in the follow-up visits.

Regarding progression from healthy elders to MCI, data indicated that, out of the 50 initially healthy participants, 33 remained healthy, while 7 developed non-AD MCI, and 10 developed MCI-AD. Among the 81 participants who developed non-AD MCI, 73 remained in this category, while 8 progressed to non-AD dementia. In contrast, among the 59 participants who developed MCI-AD, 54 progressed to AD, while only 5 remained in the MCI-AD category (Table 4 and Figure 2).

**Figure 3:**
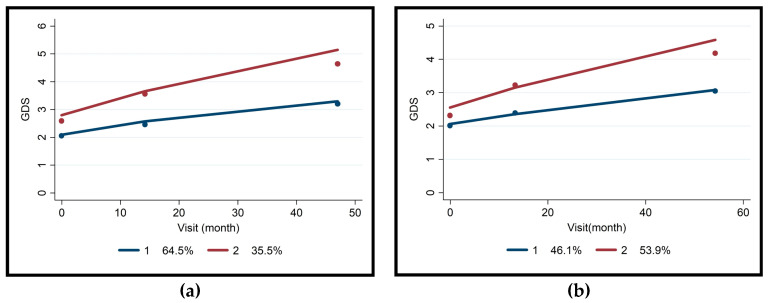
Slow/Fast prognosis of MCI (Mild Cognitive Impairment). (**a**) Slow/Fast prognosis of Non-AD MCI (Entropy = 0.833). (**b**) Slow/Fast prognosis of MCI-AD (Entropy = 0.850).

Group-based trajectory analysis identified reliable subgroups with acceptable reliability (Entropy = 0.794) among MCI cases (Table 5 and Figure 3a,b). Results showed that in the Non-AD MCI group, patients with epilepsy had a faster trajectory than those without epilepsy. Patients with hypertension in the Non-AD MCI group had a slower trajectory than those without hypertension (Figure 3a). However, no significant differences were found between the slow and fast trajectories of MCI-AD patients, except for epilepsy, where patients with epilepsy had a faster trajectory (Figure 3b). Epilepsy emerged as the most important predictor of progression (Coeff. = 2.62, *p* = 0.002).

## 4. Discussion

MCI is a complex condition with varying outcomes, including the potential for dementia. This study aimed to understand MCI progression through extensive real-life data spanning over 20 years. We found that MCI-AD progresses faster than non-AD MCI (53.9% vs. 35.5%, Entropy = 0.850), and comorbid conditions, like epilepsy, impact progression, independent of gender or established biomarkers.

Contrary to prior research, our study identified language dysfunction, especially in the MCI-AD group, as the most prevalent symptom, challenging the notion of memory dysfunction as the primary sign. Furthermore, behavioral, self-care, and incontinence issues, often reported, were less prominent in our dataset in the process of progressing through the clinical stages of dementia. This highlights the importance of early detection and intervention for language dysfunction in MCI and emphasizes the need for further research into less common symptoms. Our dataset revealed a substantial overall progression rate of 92.8% from MCI to dementia during the follow-up, equivalent to an annual rate of 15.7%. Notably, all cases of prodromal LBD/PDD and FTD progressed, while 7% of VaD and 8.4% of AD cases remained in the prodromal stage [5,15,16,17,18].

Recent studies suggest that comorbid medical conditions influence MCI progression [5,19]. Our study reinforces this link, showing that hypertension and hyperlipidemia were common comorbidities in MCI, in line with research associating them with cognitive decline and dementia [20,21]. Interestingly, our study found a low rate of regular alcohol consumption in all patient groups, aligning with evidence suggesting a potential protective role of moderate alcohol intake against cognitive decline [22,23]. Furthermore, our research highlights the significance of epilepsy and extrapyramidal symptoms in MCI progression, consistent with prior studies indicating their association with cognitive decline and dementia. The study also reveals a growing family history of vascular disease over time, linking it to cognitive decline and dementia [24,25]. These findings underscore that comorbidities significantly affect MCI progression and its subtypes, emphasizing the need for personalized treatment and management strategies.

Our investigation into cognitive function changes in MCI patients over time, using various tests, is consistent with prior research showing cognitive decline as a hallmark feature of MCI and AD [18]. The study revealed significant declines in MMSE scores and increased GDS scores, indicating overall cognitive decline and higher depression levels among participants. Furthermore, the BDLAS scores increased significantly, signifying functional capacity deterioration. The Calculation score indicated potential executive dysfunction, consistent with common cognitive deficits in MCI and AD [26]. These findings underscore the importance of tests like MMSE, GDS, and Calculation for MCI assessment (*p* < 0.0001). Significant improvements in WMT-2 and WMT-recall scores suggest that cognitive training may enhance cognitive function in MCI patients, as seen in previous studies [27]. Overall, our study provides crucial insights into cognitive function changes in MCI patients over time, emphasizing the significance of monitoring cognitive function and functional capacity to detect early signs of cognitive decline and intervene promptly. These findings, in line with prior research, affirm cognitive decline in MCI patients over time [28]. However, our study contributes by comprehensively evaluating various cognitive domains and identifying the most sensitive tests for detecting cognitive changes in MCI patients. Clinicians should consider these tests for regular monitoring of cognitive function in MCI patients, aiding in the early detection of individuals at higher risk of dementia and the need for early intervention.

The study’s results shed light on the distribution of laboratory biomarkers, hippocampal atrophy, Fazekas grading, and associated medical conditions among males and females. The prevalence of the E3/E4 genotype, followed by E3/E3, is consistent with prior studies [29,30]. Additionally, no significant differences in APOE genotype distribution between genders align with the existing literature [31]. Hippocampal atrophy grades in males and females correspond to previous findings, with most individuals having Grade 1 atrophy, followed by Grade 2. The absence of significant differences in hippocampal atrophy grades between genders is consistent with current research. Several studies have demonstrated that individuals with greater hippocampal atrophy at baseline are more likely to progress to dementia over time. For example, individuals with MCI who had greater hippocampal atrophy at baseline had a higher risk of progressing to dementia over a three-year period [32]. These findings emphasize the importance of personalized treatment and early neurodegenerative disease diagnosis [33].

The study also found no significant difference in the distribution of Fazekas grades between males and females. The majority of both genders had Grade 1, followed by Grade 2, without any effect on MCI progression trajectory. These results align with previous research suggesting that white matter hyperintensities assessed by Fazekas grading do not differ significantly between genders [34,35].

Regarding healthy individuals transitioning to MCI and MCI progressing to dementia, only a small proportion developed non-AD MCI or MCI-AD, with most staying healthy [36,37]. Among those with non-AD MCI, the majority remained in this category, with only a few progressing to non-AD dementia [38,39]. However, among those with MCI-AD, most progressed to AD, emphasizing the importance of early diagnosis and intervention [40,41].

The group-based trajectory analysis aimed to identify factors influencing cognitive impairment progression in MCI patients. Patients with Non-AD MCI and epilepsy had a faster trajectory, while those with hypertension had a slower one [42]. Epilepsy emerged as a predictor for fast progression in MCI-AD patients, and a mean GDS score level of two or below at admission correlated with slower progression [43,44]. The findings suggest that epilepsy and hypertension may play a role in the prognosis of Non-AD MCI patients, while epilepsy may predict rapid progression in MCI-AD patients. Additionally, functional disability assessed with EDLAS proved to be an important predictor of MCI progression, with cases scoring ≤21 showing fast progression [45,46]. However, the clinical application of trajectory models may be limited by data availability, especially in low- and middle-income countries. It is important to note that the slow and fast progressive subtypes of MCI may not always translate into clinically significant outcome differences [47,48].

### 4.1. Implications

The study sheds light on MCI progression, emphasizing the faster progression of MCI due to MCI-AD and the need for early detection and intervention. Notably, language dysfunction, rather than memory issues, emerges as a primary symptom in MCI, underscoring the importance of focusing on language dysfunction for early detection. The research challenges existing notions by revealing that less commonly reported symptoms, such as behavioral problems, self-care issues, and incontinence, were less prevalent even as they progressed. Comorbid medical conditions like hypertension and hyperlipidemia were common among MCI patients, warranting consideration in management. Additionally, the study highlights the significance of regular cognitive function monitoring, genetic factors, and imaging markers in MCI progression. These findings can inform healthcare professionals, potentially leading to improved diagnosis, treatment, and care for individuals with MCI. Further research is needed to refine our understanding and develop targeted interventions for different MCI subtypes and stages.

### 4.2. Limitations

The study possesses several limitations worth noting. Firstly, the sample size is relatively small, potentially limiting the generalizability of the findings. Additionally, the use of specific inclusion and exclusion criteria introduces selection bias, which may affect the sample’s representativeness. The retrospective study design is prone to recall bias and data accuracy limitations, with prospective studies being more reliable. Conducting the research in a single center may limit its applicability to diverse populations. Despite efforts to control confounding factors, there may be unaccounted variables influencing MCI progression. Furthermore, the assessment tools used might not capture the full spectrum of cognitive impairment or underlying pathology. Notably, there was a lack of detailed neuropsychiatric evaluation, which could have provided valuable insights into the cognitive and psychiatric aspects of MCI. The study solely focused on natural MCI progression, omitting the evaluation of interventions or treatments. Reliance on self-reported symptoms and comorbidities may introduce recall bias or subjective interpretations. Lastly, the study did not conduct detailed subgroup analyses based on factors such as age, education, or socioeconomic status, which could influence MCI progression. Additionally, we excluded overlapping syndromes and mixed dementia, acknowledging this as a limitation in the manuscript, and its potential impact on the findings should be considered.

## 5. Conclusions

In conclusion, this study provides valuable insights into the characteristics and progression of participants with different subtypes of MCI. The study found that participants with MCI-AD and Non-AD MCI had a higher mean age compared to the healthy control group, with the Non-AD MCI group being the oldest. Language dysfunction and memory dysfunction were the most common symptoms reported, with hypertension and hyperlipidemia being the most common comorbidities. Cognitive function declined over time, with MMSE scores showing a significant decline from the first to the third visit. The study also found that there were significant improvements in WMT-2 and WMT-recall scores over the three visits. The APOE genotype distribution, hippocampal atrophy grades, and associated medical conditions were also reported. The study further found that epilepsy and hypertension may have an impact on the prognosis of Non-AD MCI patients, while epilepsy may be a predictor for the fast progression of MCI-AD patients. Overall, these findings contribute to a better understanding of MCI and can aid in the development of targeted interventions for patients with MCI.

## Figures and Tables

**Table 1:** Demographic and clinical features of study sample.

	Healthy Control n = 50 (26.31%)	MCI-AD n = 59 (31.05%)	Non-AD MCI n = 81 (42.63%)	Total MCI n = 140 (73.68%)	Total n = 190 (100%)	*p*
Age. year (mean ± SD)	65.59 ± 9.96	69.51 ± 6.32	71.12 ± 7.32	70.44 ± 6.94	69.26 ±8.07	0.0015
Follow-up (months)						
(mean ± SD) (min–max)	28.32 ± 35.11	27.05 ± 25.19	28.63 ± 27.84	27.96 ± 26.68	38.06 ± 29.03	0.948683
First visit *	(0–120)	(3–120)	(3–120)	(3–120)	(0–120)	
	-	12.95 ± 11.92	15.06 ± 13.71	14.17 ± 12.98	14.17 ± 12.98	0.343653
Second visit		(3–60)	(3–79)	(3–79)	(3–79)	
	26.72 ± 27.58	41.03 ± 37.68	31.38 ± 31.63	35.45 ± 34.51	33.15 ± 32.99	0.063143
Last visit **	(4–127)	(3–174)	(2–137)	(12–203)	(2–174)	
Gender						
Female n (%)	28 (56%)	31 (52.54%)	47 (58.02%)	78 (55.71%)	106 (55.79%)	0.81173
Male n (%)	22 (44%)	28 (47.46%)	34 (41.98%)	62 (44.28%)	84 (44.21%)	
Formal education, n (%)						
Basic school	42 (84%)	47 (79.66%)	73 (90.12%)	120 (85.71%)	162 (85.26%)	0.14378
Middle school	-	2 (3.39%)	2 (2.47%)	4 (2.86%)	4 (2.10%)	
High school	-	2 (3.38%)	3 (3.70%)	5 (3.57%)	5 (2.63%)	
University	8 (16%)	8 (13.56%)	3 (3.70%)	11 (7.86%)	19 (10%)	
Living n (%)						
Alone	3 (6%)	8 (13.56%)	15 (18.52%)	23 (16.43%)	26 (13.68%)	0.12854
with family	47 (94%)	51 (86.44%)	66 (81.48%)	117 (83.57%)	164 (86.32%)	
Presentation symptoms n (%)						
Memory dysfunction		26 (44.07%)	25 (30.86%)	51 (36.43%)	51 (26.84%)	0.19237
Language dysfunction		38 (64.41%)	47 (58.02%)	85 (60.71%)	44 (74%)	0.14820
Executive dysfunction		8 (13.56%)	11 (13.58%)	19 (13.57%)	19 (10%)	0.02306
Behavioral problem		1 (1.69%)	1 (1.23%)	2 (1.43%)	2 (1.05%)	0.67323
Self-Care problem		3 (5.08%)	4 (4.94%)	7 (5%)	7 (3.68%)	0.96302
Sleep problem		21 (35.59%)	20 (24.69%)	41 (29.28%)	41 (21.58%)	0.31678
Disorientation		12 (20.34%)	21 (25.93%)	33 (23.57%)	33 (17.37%)	0.52651
Incontinence		8 (13.56%)	20 (24.69%)	28 (20%)	28(14.74%)	0.06346
Loss of appetite		7 (11.86%)	9 (11.11%)	16 (11.43	16 (8.42%)	0.95305
Comorbid medical problems n (%)						
Hypertension	19 (38%)	22 (37.29%)	38 (46.92%)	60 (42.86%)	79 (41.58%)	0.43609
Thyroid dysf.	7 (14%)	10 (16.95%)	12 (14.81%)	22 (15.71%)	29 (15.26%)	0.90305
Diabetes Mellitus	9 (18%)	11 (18.64%)	19 (23.46%)	30 (21.43%)	39 (20.52%)	0.68723
CAD	12 (24%)	15 (25.42%)	23 (28.40%)	38 (27.14%)	50 (26.31%)	0.84236
Hyperlipidemia	9 (18%)	14 (23.73%)	20 (24.69%)	34 (24.28%)	43 (22.63%)	0.65397
Stroke	1 (2%)	1 (1.69%)	5 (6.17%)	6 (4.28%)	7 (3.68%)	0.29063
Current Smoker	7 (14%)	4 (6.78%)	14 (17.28%)	18 (12.86%)	25 (13.16%)	0.44638
No smoker	32 (64%)	41 (69.49%)	47 (58.02%)	88 (62.86%)	120 (63.16)	
Ex-smoker	11 (22%)	14 (23.73%)	20 (24.69%)	34 (24.28%)	45 (23.68%)	
Regular alcoholic	-	9 (15.25%)	6 (7.41%)	15 (10.72%)	15 (7.89%)	0.29225
Non-alcoholic	47 (94%)	49 (83.05%)	73 (90.12%)	122 (87.14%)	169 (88.95%)	
Ex-alcoholic	3 (6%)	1 (1.69%)	2 (2.47%)	3 (2.14%)	6 (3.16%)	
Epilepsy	-	4 (6.78%)	14 (17.28%)	18 (12.86%)	18 (9.47%)	**0.00319**
Extrapyramidal symptoms	-	1 (1.69%)	18 (22.22%)	19 (13.57%)	19 (10%)	**0.00004**
Family history of dementia n (%) Family history of vascular disease	18 (36%)	22 (37.29%)	39 (48.15%)	61 (43.57%)	79 (41.58%)	0.28268
	23 (46%)	30 (50.85%)	43 (53.09%)	73 (52.14%)	96 (50.53%)	0.73176

* First visit duration means the duration of the first presenting symptom. dysf: dysfunction. ** Total follow-up duration is 71.65 ± 44.82 months for (min 6 to max 203 months) (n = 190).

**Table 2:** Neuropsychiatric evaluation of the cases with MCI (n = 140).

	First Visit	Second Visit	Third Visit	*p*
BDLAS mean ± SD (min–max)	1.20 ± 0.99 (0–4.5)	1.63 ± 1.10 (0–6)	2.33 ± 1.64 (0–7.5)	0.00042 *
EDLAS mean ± SD (min–max)	21.28 ±3.07 (8–23)	19.78 ± 4.63 (3–23)	16.70 ± 6.86 (0–23)	**0.00663**
MMSE mean ± SD (min–max)	27.28 ± 2.66 (15–30)	26.74 ± 3.42 (13–30)	23.06 ± 6.34 (3–30)	<0.0001 *
Digit forward: median (min–max)	4 (2–7)	4 (3–7)	4 (0–7)	0.82657
Digit backward: median (min–max)	3 (0–5)	3 (0–6)	3 (0–7)	0.18348
Calculation: median (min–max)	5 (0–5) n = 128	5 (0–5) n = 127	4 (0–5) n = 127 (0–5)	<0.0001 *
Abstraction: median (min–max)	3 (0–3) n = 131	3 (0–3) n = 130	3 (0–3) n = 130	0.00009 *
WMT-1: median (min–max)	3 (0–7)	3 (0–10)	3 (0–7)	**0.03915**
	n = 124	n = 127	n = 127	
WMT-2: median (min–max)	5 (0–8)	4 (0–10)	4 (0–8)	0.00146 *
	n = 125	n = 127	n = 128	
WMT-3: median (min–max)	5 (0–10)	5 (0–9)	5 (0–9)	**0.02696**
	n = 126	n = 126	n = 128	
WMT-recall: median (min–max)	3 (0–10)	3 (0–8)	2 (0–9)	**0.00529**
	n = 122	n = 123	n = 124	
WMT-recognition: median (min–max)	17 (0–20)	18 (0–20)	16 (0–20)	**0.00362**
	n = 122	n = 126	n = 127	
BNT: median (min–max)	14 (3–15) n = 128	14 (2–15) n = 124	13 (0–15) n = 130	0.00186 *
CDT: median (min–max)	9 (0–10) n = 121	10 (0–10) n = 123	8 (0–10) n = 123	0.12589
GDS: median (min–max)	2 (1–3) n = 140	3 (2–5) n = 140	3 (2–7) n = 140	<0.0001 *
Comprehension: median (min–max)	6 (1–6) n = 77	6 (1–6) n = 87	6 (0–6) n = 90	**0.04630**
Visual memory score: median (min–max)	11 (0–11) n = 48	10 (0–11) n = 49	8 (0–11) n = 70	0.07971
Visual memory-recall: median (min–max)	8 (0–11) n = 47	7 (0–11) n = 47	3 (0–11) n = 67	0.52839

Abbreviations: BDLAS (Barthel Daily Living Activities Scale), EDLAS (Elderly Daily Living Activity Scale), MMSE (Mini-mental State Examination), WMT (Word Memory Test), BNT (Boston Naming Test), CDT (Clock Driving Test), GDS (Global Deterioration Scale). *: Statistically significant according to Bonferroni adjusted *p* value (0.0027). “Values in bold indicate that they are significant *p* values”.

**Table 3:** Laboratory evaluation of the cases with MCI.

n (%)	Male (n = 62)	Female (n = 78)	Total (n = 140)	*p*
APOE genotype				0.38171
E3/E4	6 (66.67%)	5 (55.56%)	11 (61.12%)	
E3/E3	3 (33.33%)	2 (22.22%)	5 (27.78%)	
E2/E4	0 (0%)	1 (11.11%)	1 (5.55%)	
E4/E4	0 (0%)	1 (11.11%)	1 (5.55%)	
Hippocampal atrophy n (%)				0.44000
Grade 1	32 (52.46%)	49 (62.82%)	81 (58.27%)	
Grade 2	25 (40.98%)	24 (30.77%)	49 (35.26%)	
Grade 3	4 (6.56%)	5 (6.41%)	9 (6.47)	
Fazekas grading n (%)				0.47120
Grade 0	9 (15.25%)	19 (25.33%)	28 (20.90%)	
Grade 1	23 (38.98%)	29 (38.67%)	52 (38.81%)	
Grade 2	19 (32.20%)	20 (26.67%)	39 (29.10%)	
Grade 3	8 (13.56%)	7 (9.33%)	15 (11.19%)	
Associated				
Hydrocephalus	0 (0%)	0(0%)	0 (0%)	N.A.
Epilepsy	8 (12.90%)	10 (12.82%)	18 (12.86%)	0.98841
Extrapyramidal symptoms	12 (19.35%)	7 (8.97%)	19 (13.57%)	0.07485

**Table 4:** Progression of the cases in the follow-up visits.

	Study Groups					
	Healthy	NonAD-MCI	MCI-AD	Non-AD	AD	Row Totals
Healthy	33	7	10	0	0	50
Non-AD MCI	0	8	0	73	0	81
MCI-AD	0	0	5	0	54	59
Totals	33	15	15	73	54	190

**Table 5:** Observed variables on the prognosis of Non-AD MCI and MCI-AD *.

	Non-AD MCI (n = 81)				MCI-AD (n = 59)			
	Slow	Fast	Trajectory Model		Slow	Fast	Trajectory Model	
	n = 52 (64.5%)	n = 29 (35.5%)	Coeff. ± Std. Error	*p*	n = 27 (46.1%)	n = 32 (53.9%)	Coeff. ± Std. Error	*p*
Fazekas grading			0.29 ± 0.33	0.387			−0.106 ± 0.41055	0.794
0	6	1			8	13		
1	16	7			13	14		
2	19	11			4	3		
3	7	4			0	1		
Hippocampal atrophy			0.74 ± 0.47	0.116			0.856 ± 0.50845	0.094
1	38	15			12	10		
2	9	10			13	16		
3	1	1			0	5		
BDLAS-1	0.98 ± 0.75 (0–3.5)	1.69 ± 1.22 (0–4.5)	0.16 ± 0.52	0.751	0.95 ± 0.96 (0–3)	1.33 ± 0.98 (0–3)	0.44 ± 0.33	0.180
BDLAS-2	NA	NA			NA	NA		
BDLAS-3	NA	NA			NA	NA		
EDLAS-1	22.22 ± 1.5 (16–23)	19 ± 4.6 (8–23)	−0.4 ± 0.2	0.065	22.0 ± 1.3 (19–23)	20.9 ± 3.3 (10–23)	−0.2 ± 0.1	0.168
EDLAS-2	21.03 ± 3.37 (13–23)	18.27 ± 5.11 (3–23)			19.84 ± 5.84 (6–23)	19.578 ± 4.58 (10–23)		
EDLAS-3	20.73 ± 3.01 (13–23)	12.684 ± 7.93 (0–23)			20.06 ± 4.18 (0–23)	13.45 ± 7.11 (10–23)		
Epilepsy			2.62 ± 0.86	**0.002**			**NA**	**NA**
No	44	16			24	28		
Yes	4	10			1	3		
Extrapyramidal symptoms			−0.48 ± 0.91	0.594			NA	NA
No	37	21			25	30		
Yes	11	5			0	1		
Thyroid dysfunction			0.13 ± 0.86	0.879			0.17 ± 0.82	0.833
No	41	23			21	25		
Yes	7	3			4	6		
Coronary artery disease			0.53 ±0.76	0.484			−0.610 ± 0.71777	0.396
No	37	18			17	24		
Yes	11	8			8	7		
Diabetes mellitus			0.10 ± 0.90	0.910			0.35 ± 0.80	0.662
No	39	18			21	24		
Yes	9	8			4	7		
Hypertension			1.33 ± 0.77	0.084			0.87 ± 0.70	0.216
No	28	10			18	17		
Yes	20	16			7	14		
Stroke			0.67 ± 1.37	0.624			NA	NA
No	47	24			25	30		
Yes	1	2			0	1		
Hyperlipidemia			0.26 ± 0.85	0.753			0.66 ± 0.73	0.367
No	37	19			20	22		
Yes	11	7			5	9		
Sleep disorders			−0.09 ± 0.80	0.903			0.45 ± 0.67	0.503
No	11	7			18	18		
Yes	37	19			7	13		
Smoking			−0.07 ± 0.41	0.857			0.94 ± 0.61	0.126
Current	8	5			3	0		
None	28	16			17	23		
Quit	12	5			5	8		
Regular Alcohol usage			−0.29 ± 0.92	0.750			0.03 ± 0.82	0.963
Current	2	4			5	3		
None	46	21			19	28		
Quit	0	1			1	0		
Family history of dementia			0.19 ± 0.54	0.721			−0.56 ± 0.59	0.340
No	27	12			14	21		
Yes	21	14			11	10		

Abbreviations: BDLAS (Barthel Daily Living Activities Scale), EDLAS (Elderly Daily Living Activity Scale). * Regarding some missing data: some of the test total count of samples are not the same in the table.

## Data Availability

The data analyzed in this study is subject to the following licenses/restrictions: this is only accessible to doctors who are members of the system. Requests to access these datasets should be directed to http://www.epikriz.com/index.aspx (accessed on 3 January 2024).
